# Correction: Patient-derived oral mucosa organoids as an *in vitro* model for methotrexate induced toxicity in pediatric acute lymphoblastic leukemia

**DOI:** 10.1371/journal.pone.0237488

**Published:** 2020-08-05

**Authors:** E. Driehuis, N. Oosterom, S. G. Heil, I. B. Muller, M. Lin, S. Kolders, G. Jansen, R. de Jonge, R. Pieters, H. Clevers, M. M. van den Heuvel-Eibrink

## Notice of republication

An incorrect version of S1 Fig was published in error. This article was republished on July 27, 2020 to correct for this error. Please download this article again to view the correct version.

## References

[1] Driehuis E, Oosterom N, Heil SG, Muller IB, Lin M, Kolders S, et al (2020) Patient-derived oral mucosa organoids as an *in vitro* model for methotrexate induced toxicity in pediatric acute lymphoblastic leukemia. PLoS ONE 15(5): e0231588 10.1371/journal.pone.0231588 32421698 PMC7233536

