# Supplementary figures and images for: Patient-derived oral mucosa organoids as an in vitro model for methotrexate induced toxicity in pediatric acute lymphoblastic leukemia
Source: PLoS One. 2020 May 18;15(5):e0231588. doi: 10.1371/journal.pone.0231588 (PMC7233536; doi:10.1371/journal.pone.0231588)

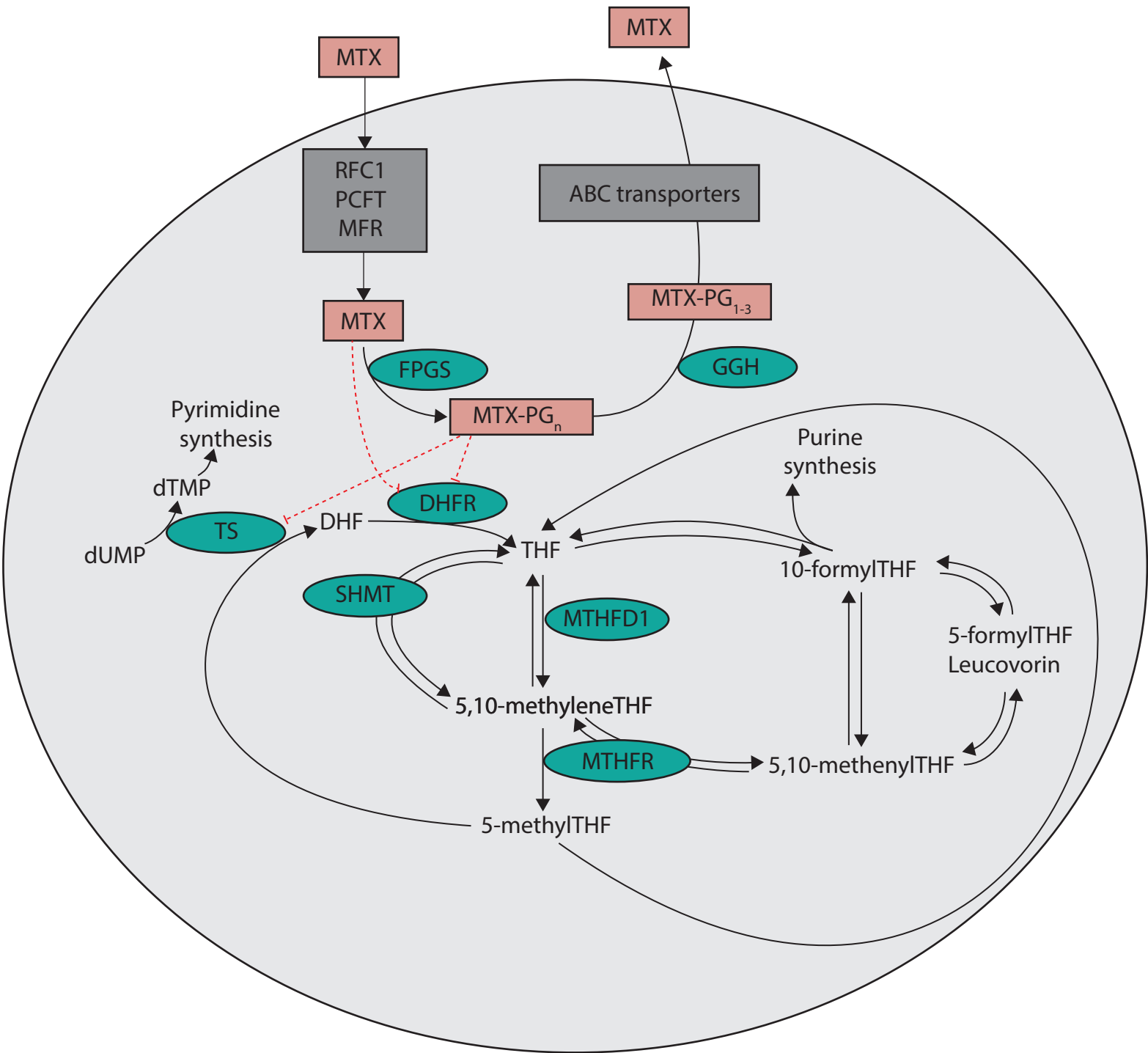

Supplement: S1 Fig — Leucovorin (5-formylTHF) is represented in bold / italic. MTX enters the cell mainly through the Reduced Folate Carrier 1 (RFC1), Proton Coupled Folate Transporter (PCFT), Membrane Folate Transporters (MFR) or by passive diffusion through the cell membrane. While circulating, MTX contains one polyglutamate group (MTX-PG1). Once inside the cell, MTX is polyglutamated by Folylpolyglutamate Synthetase (FPGS) with up to seven polyglutamate groups. Long-chain MTX-PG’s (MTX-PG4-7) cannot be transported out of the cell before de-polyglutamation by Gamma-Glutamyl Hydrolase (GGH). Short-chain MTX-PG’s (MTX-PG1-3) will be actively transported out of the cell by ABCC1-4, ABCB1 and ABCG2 transporters. MTX is cytotoxic as it impairs purine- and pyrimidine synthesis by inhibiting the enzymes Dihydrofolate Reductase (DHFR) and Thymidylate Synthase (TYMS). Abbreviations: ABCB1—ATP Binding Cassette Subfamily B Member 1; ABCC1-4—ATP Binding Cassette Subfamily C Member 1–4; ABCG2—ATP Binding Cassette Subfamily G Member 2; DHFR–Dihydrofolate Reductase; FPGS–Folylpolyglutamate Synthetase; GGH–Gamma-Glutamyl Hydrolase; MFR–Membrane Folate Transporter; MTHFR—Methylene tetrahydrofolate reductase; MTHFD1—Methylenetetrahydrofolate Dehydrogenase, Cyclohydrolase And Formyltetrahydrofolate Synthetase 1; PCFT–Proton-Coupled Folate Transporter; RFC1 –Reduced Folate Carrier; SHMT—Serine hydroxymethyltransferase; TS–Thymidylate Synthase. (PDF) [file pone.0231588.s001.pdf]
