# Supplementary material for: Patient-derived oral mucosa organoids as an in vitro model for methotrexate induced toxicity in pediatric acute lymphoblastic leukemia
Source: PLoS One. 2020 May 18;15(5):e0231588. doi: 10.1371/journal.pone.0231588 (PMC7233536; doi:10.1371/journal.pone.0231588)
Supplement: S2 Fig — A. Oral mucosa organoids are derived of human normal cells, and not cancer cells. Number of mutations detected by whole exome sequencing in the healthy oral mucosa organoids used in this study, and their corresponding tumor organoids. Mutational load is low (2 for N1, 0 for T1), especially when compared to the tumor organoids. B. FPGS activity (in pmol MTX-PG2/h/mg) in organoid line versus CCRF-CEM reference leukemia cell line. C. Effect of PT on MTX-PG levels in oral mucosa organoid lines derived from two different donors. D. Effect of PT on MTX-PG levels in two B-ALL and two T-ALL cell lines. (PDF) [file pone.0231588.s002.pdf]

Figure S2. Characterization of oral mucosa organoids and the effect of pretreatment on intracellular MTX-PG levels.

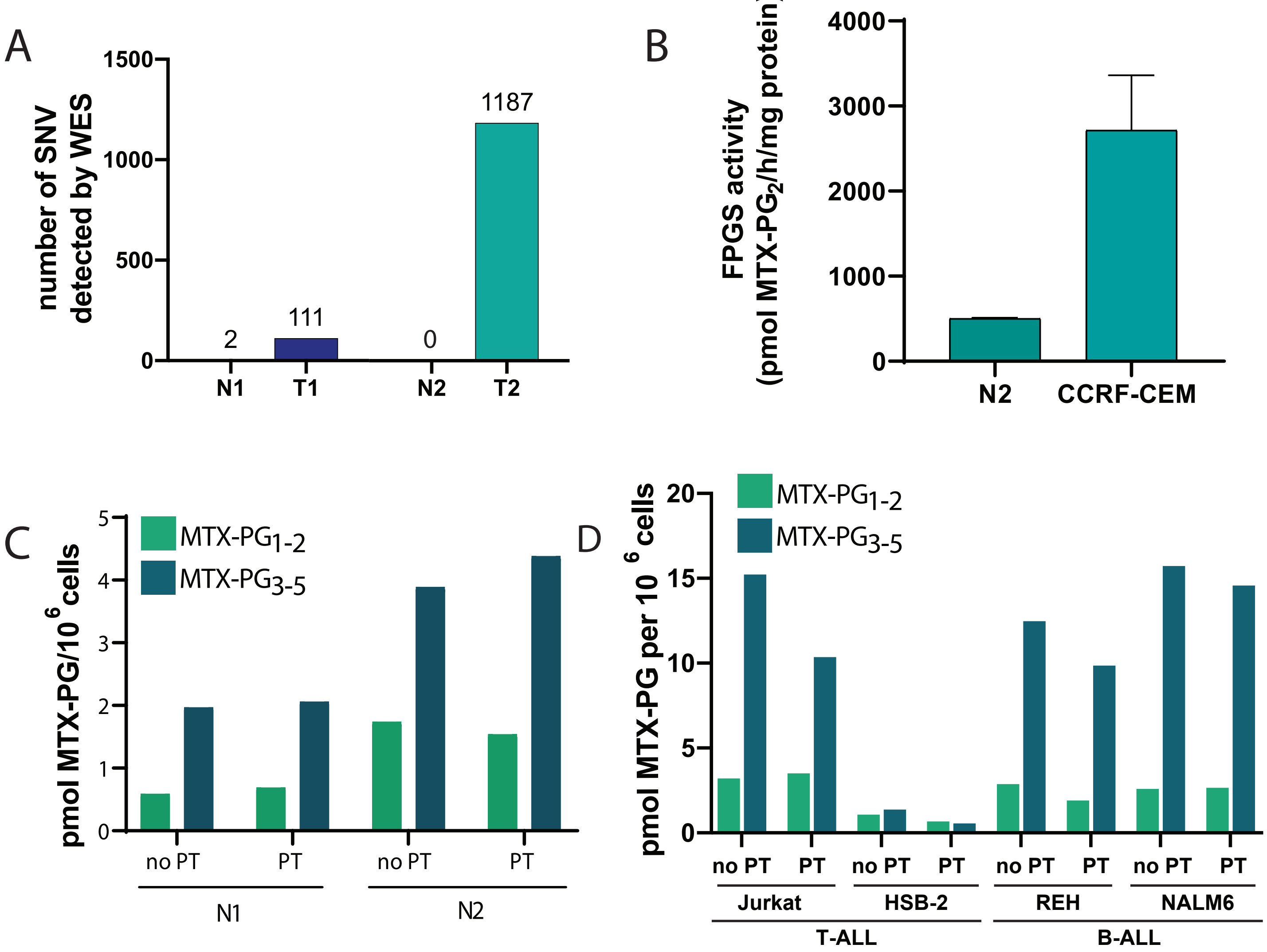

!"#$#%&'$()#&(*+,*-,*$#.,/0%*1#,*$2#+*(31,#+3,&"','--'%&,*-,4$'&$'#&/'+&,*+,(+&$#%'..0.#$,567 89:,
.' ;'.1<,!
