# Supplementary material for: Patient-derived oral mucosa organoids as an in vitro model for methotrexate induced toxicity in pediatric acute lymphoblastic leukemia
Source: PLoS One. 2020 May 18;15(5):e0231588. doi: 10.1371/journal.pone.0231588 (PMC7233536; doi:10.1371/journal.pone.0231588)

Figure S3. Oral mucosa organoids grown in folate-deprived medium grow comparable to organoids grown in complete medium.

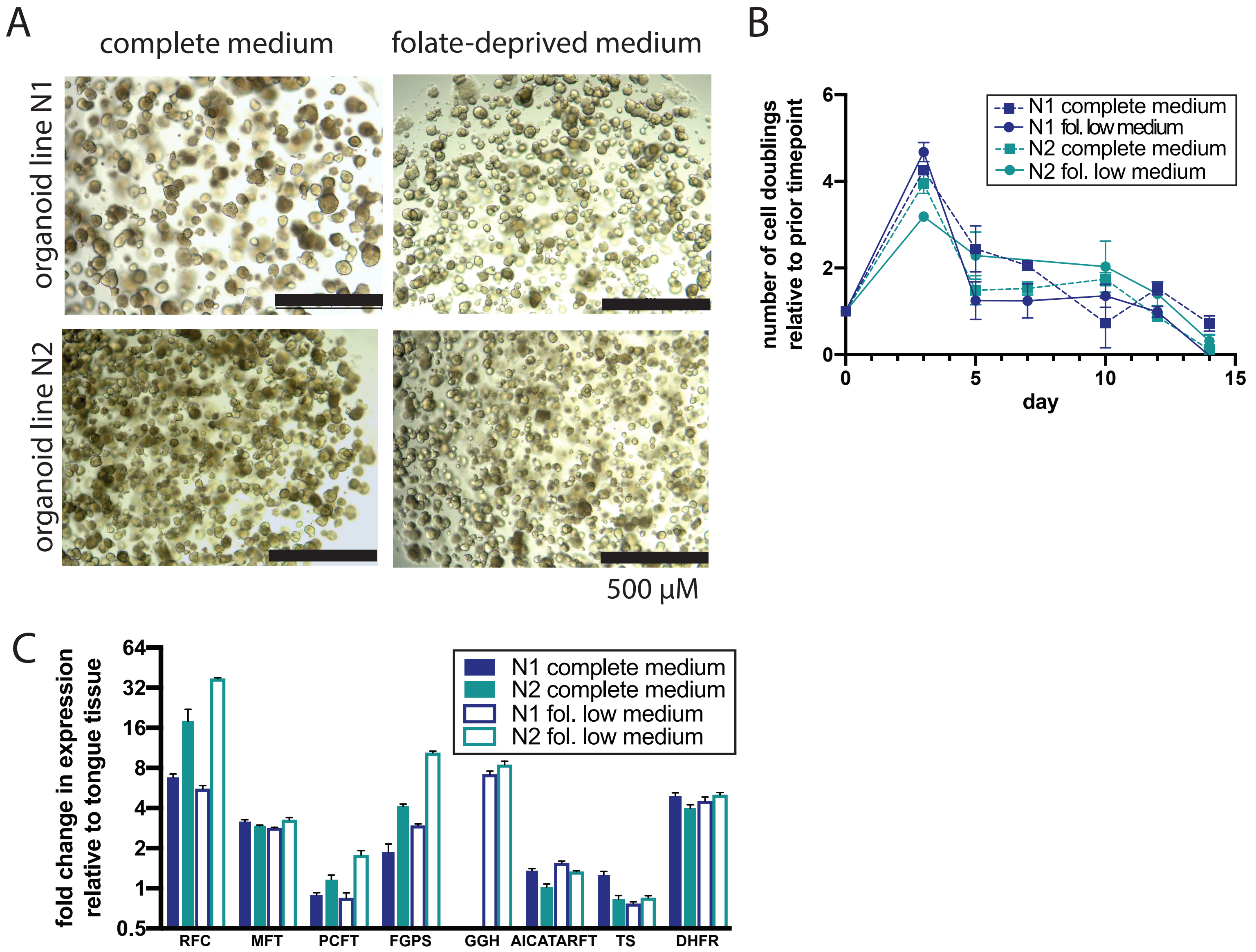

Supplement: S3 Fig — A. Brightfield microscopy images of organoid line N1 and N2, when grown in either complete medium, or folate deprived medium. Scalebar, 500 μm. B. Growth speed of organoid cultures in both media tested. Growth was assessed by collection of cell pellets at day 0, 3, 5, 7, 10 and 14. Cell number was assessed by cell titer glow and values were made relative to day 0. C. Quantitative PCR assessing expression of genes relevant for methotrexate metabolism. Experiment was performed in triplicate, results of all three experiments are shown here. (PDF) [file pone.0231588.s003.pdf]
