# Supplementary material for: Patient-derived oral mucosa organoids as an in vitro model for methotrexate induced toxicity in pediatric acute lymphoblastic leukemia
Source: PLoS One. 2020 May 18;15(5):e0231588. doi: 10.1371/journal.pone.0231588 (PMC7233536; doi:10.1371/journal.pone.0231588)

Figure S4. Technical details of drug screens performed in this study

A

MTX range concentrations: 5 - 0.05 µM in triplicate

<>	1	2	3	4	5	6	7	8	9	10	11	12	13	14	15	16	17	18	19	20	21	22	23	24
A																								
B	Block 1	5,00	2,99	1,79	1,09	0,64	0,38	0,22	0,13	0,08	0,05	Staurosporine	5,00	2,99	1,79	1,09	0,64	0,38	0,22	0,13	0,08	0,05	Block 5	
C	MTX only	5,00	2,99	1,79	1,09	0,64	0,38	0,22	0,13	0,08	0,05		5,00	2,99	1,79	1,09	0,64	0,38	0,22	0,13	0,08	0,05	+ LV T48h	
D		5,00	2,99	1,79	1,09	0,64	0,38	0,22	0,13	0,08	0,05		5,00	2,99	1,79	1,09	0,64	0,38	0,22	0,13	0,08	0,05		
E	Block 2	5,00	2,99	1,79	1,09	0,64	0,38	0,22	0,13	0,08	0,05	Blanco	5,00	2,99	1,79	1,09	0,64	0,38	0,22	0,13	0,08	0,05	Block 6	
F	+ LV T0h	5,00	2,99	1,79	1,09	0,64	0,38	0,22	0,13	0,08	0,05		5,00	2,99	1,79	1,09	0,64	0,38	0,22	0,13	0,08	0,05	+ LV T72h	
G		5,00	2,99	1,79	1,09	0,64	0,38	0,22	0,13	0,08	0,05		5,00	2,99	1,79	1,09	0,64	0,38	0,22	0,13	0,08	0,05		
H	Block 3	5,00	2,99	1,79	1,09	0,64	0,38	0,22	0,13	0,08	0,05		5,00	2,99	1,79	1,09	0,64	0,38	0,22	0,13	0,08	0,05	Block 7	
I	+ LV T12h	5,00	2,99	1,79	1,09	0,64	0,38	0,22	0,13	0,08	0,05		5,00	2,99	1,79	1,09	0,64	0,38	0,22	0,13	0,08	0,05	+ LV T96h	
J		5,00	2,99	1,79	1,09	0,64	0,38	0,22	0,13	0,08	0,05		5,00	2,99	1,79	1,09	0,64	0,38	0,22	0,13	0,08	0,05		
K	Block 4	5,00	2,99	1,79	1,09	0,64	0,38	0,22	0,13	0,08	0,05													
L	+ LV T24h	5,00	2,99	1,79	1,09	0,64	0,38	0,22	0,13	0,08	0,05													
M		5,00	2,99	1,79	1,09	0,64	0,38	0,22	0,13	0,08	0,05													
N																								
O																								
P																								

B

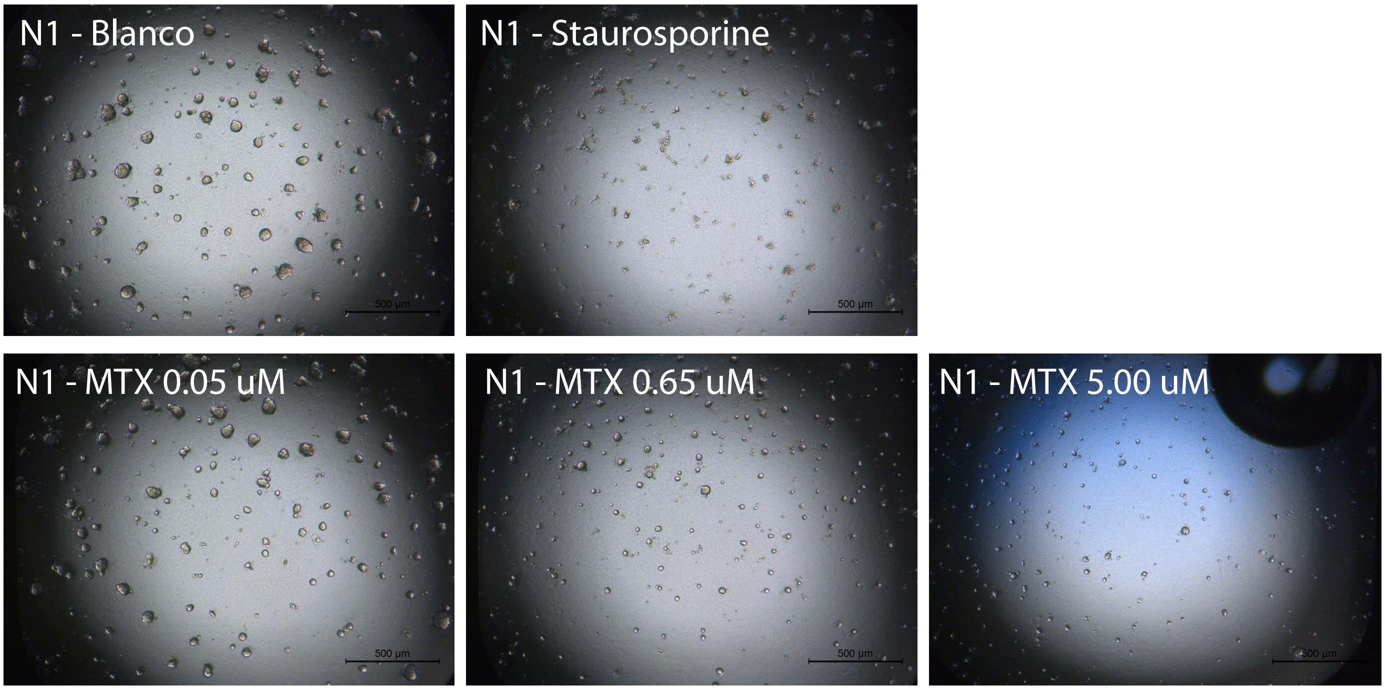

C

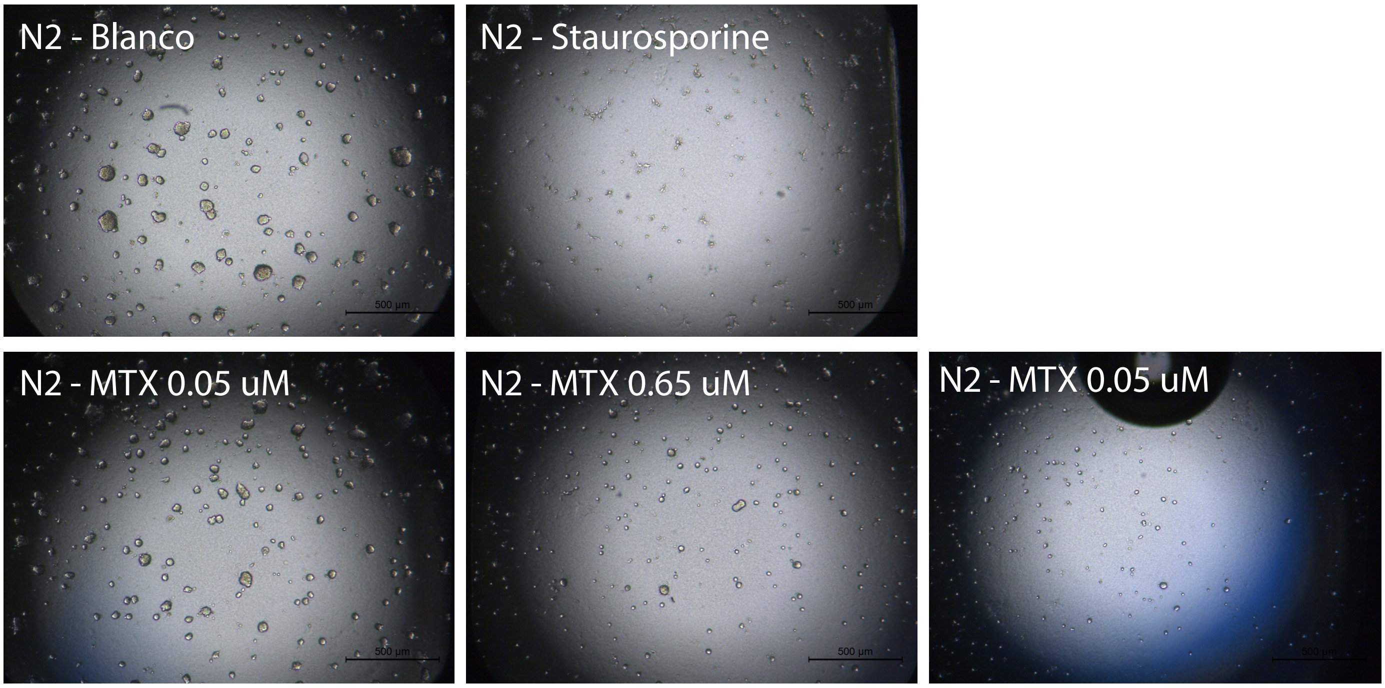

Supplement: S4 Fig — A. Schematic layout of a drug screen plate as used in this study. The gradient of MTX is depicted using a color gradient (red indicates high concentration, green indicates low concentration). Here, the MTX concentrations used for organoids are depicted. Each concentration is tested in technical triplicate. Different blocks receive LV rescue at different timepoints after the start of MTX treatment, as indicated. Staurosporine treated wells are used as positive controls and are set to 0% viability, wells only receiving drug solvent are used is negative controls, and are set to 100% viability. B. Brightfield microscopy images showing the morphology of N1 organoids in drug screening plates on the day of readout. C. Brightfield microscopy images showing the morphology of N2 organoids in drug screening plates on the day of readout. (PDF) [file pone.0231588.s004.pdf]
