# Supplementary material for: Patient-derived oral mucosa organoids as an in vitro model for methotrexate induced toxicity in pediatric acute lymphoblastic leukemia
Source: PLoS One. 2020 May 18;15(5):e0231588. doi: 10.1371/journal.pone.0231588 (PMC7233536; doi:10.1371/journal.pone.0231588)
Supplement: S1 Table — (PDF) [file pone.0231588.s005.pdf]

# Table S1. Clinical information of patients

| name | gender | age at diagnosis | tumor location |
|---|---|---|---|
| N1 | male | 80 | parotis SCC |
| N2 | female | 70 | gingiva |
| N3 | male | 68 | larynx |
| N4 | male | 65 | salivary gland SCC |
| N5 | female | 60 | oral cavity |
