# Supplementary material for: Patient-derived oral mucosa organoids as an in vitro model for methotrexate induced toxicity in pediatric acute lymphoblastic leukemia
Source: PLoS One. 2020 May 18;15(5):e0231588. doi: 10.1371/journal.pone.0231588 (PMC7233536; doi:10.1371/journal.pone.0231588)
Supplement: S2 Table — (PDF) [file pone.0231588.s006.pdf]

S2 Table

Organoid line / Leukemia cell line	Z-scores			
N1	0,6465	0,7194	0,4499	0,3539
N1 - PT	0,4956	0,6276	0,3304	0,8192
N2	0,3644	0,8923	0,8424	0,4888
N2 - PT	0,5608	0,7385	0,7172	0,5921
N3	0,7360			
N3 - PT	0,9172			
N4	0,6484			
N4 - PT	0,6074			
N5	0,9296			
N5 - PT	0,4788			
Jurkat	0,9285	0,8396	0,8985	
Jurkat - PT	0,9782	0,9413	0,9545	
NALM6	0,8172	0,7597	0,5932	
NALM6 - PT	0,8708	0,9015	0,6629	
REH	0,9743	0,7067	0,8949	
REH - PT	0,8998	0,8019	0,6656	
HSB2	0,9202	0,9193	0,8629	
HSB2 - PT	0,9312	0,8975	0,8973	
MOLT16	0,8955	0,9439	0,7531	
MOLT16 - PT	0,9415	0,8294	0,6990	
**Median**	0,8182			
**Minimum range**	0,3304			
**Maximum range**	0,9782			
