# Supplementary material for: Patient-derived oral mucosa organoids as an in vitro model for methotrexate induced toxicity in pediatric acute lymphoblastic leukemia
Source: PLoS One. 2020 May 18;15(5):e0231588. doi: 10.1371/journal.pone.0231588 (PMC7233536; doi:10.1371/journal.pone.0231588)
Supplement: S4 Table — 5’ to 3’ sequences of primers used to assess gene expression by quantitative PCR in this study. (PDF) [file pone.0231588.s008.pdf]

Table S4. Sequences of primers used for quantitative PCR

primer name	primer sequence	length of product
PCFT RT F	5'-CACTCTACCCAGCCACTCTGAAC-3'	127 bp
PCFT RT R	5'-GATCAGCCTTTTCCAGCATCC-3'	
RFC F	5'-ACCATCATCACTTTCATTGTCTC-3'	97 bp
RFC R	5'-ATGGACAGGATCAGGAAGTACA-3'	
MFT F	5'-GCCGTGAGTGATGGATTGGAA-3'	102 bp
MFT R	5'-TCCTTGATAAAGTCCCCGTAGT-3'	
DHFR F	5'-ATGCCTTAAAACTTACTGAACAACCA-3'	81 bp
DHFR R	5'-TGGGTGATTCATGGCTTCCT-3'	
TS F	5'-TCCCGAGACTTTTTGGACAGC-3'	166 bp
TS R	5'-TGATGGTGTCAATCACTCTTTGC-3'	
AICARTF F	5'-ACCTGACCGCTCTTGGTTTG-3'	171 bp
AICARTF R	5'-TACGAGCTAGGATTCCAGCAT-3'	
FPGS F	5'-CCGAGCATGGAGTACCAGGA-3'	80 bp
FPGS R	5'-GCGCTTCACCTGCTCCAG-3'	
GGH F	5'-GCGAGAGTTGTACCAGTAAGGC-3'	118 bp
GGH R	5'-CATAATCTGAGCGTCTGAGGTC-3'	
